# Plasma GFAP, NfL and pTau 181 detect preclinical stages of dementia

**DOI:** 10.3389/fendo.2024.1375302

**Published:** 2024-04-09

**Authors:** Assunta Ingannato, Silvia Bagnoli, Salvatore Mazzeo, Giulia Giacomucci, Valentina Bessi, Camilla Ferrari, Sandro Sorbi, Benedetta Nacmias

**Affiliations:** ^1^ Department of Neuroscience, Psychology, Drug Research and Child Health, University of Florence, Florence, Italy; ^2^ Research and Innovation Centre for Dementia-CRIDEM, Azienda Ospedaliero-Universitaria Careggi, Florence, Italy; ^3^ Vita-Salute San Raffaele University, Milan, Italy; ^4^ Istituti di Ricovero e Cura a Carattere Scientifico (IRCCS) Policlinico San Donato, San Donato Milanese, Italy; ^5^ Istituti di Ricovero e Cura a Carattere Scientifico (IRCCS) Fondazione Don Carlo Gnocchi, Florence, Italy

**Keywords:** Alzheimer's disease, preclinical stages, plasma biomarkers, glial fibrillary acidic protein, neurofilament light chain, phosphorylated-tau-181

## Abstract

**Background:**

Plasma biomarkers are preferable to invasive and expensive diagnostic tools, such as neuroimaging and lumbar puncture that are gold standard in the clinical management of Alzheimer’s Disease (AD). Here, we investigated plasma Glial Fibrillary Acidic Protein (GFAP), Neurofilament Light Chain (NfL) and Phosphorylated-tau-181 (pTau 181) in AD and in its early stages: Subjective cognitive decline (SCD) and Mild cognitive impairment (MCI).

**Material and methods:**

This study included 152 patients (42 SCD, 74 MCI and 36 AD). All patients underwent comprehensive clinical and neurological assessment. Blood samples were collected for Apolipoprotein E (APOE) genotyping and plasma biomarker (GFAP, NfL, and pTau 181) measurements. Forty-three patients (7 SCD, 27 MCI, and 9 AD) underwent a follow-up (FU) visit after 2 years, and a second plasma sample was collected. Plasma biomarker levels were detected using the Simoa SR-X technology (Quanterix Corp.). Statistical analysis was performed using SPSS software version 28 (IBM SPSS Statistics). Statistical significance was set at p < 0.05.

**Results:**

GFAP, NfL and pTau 181 levels in plasma were lower in SCD and MCI than in AD patients. In particular, plasma GFAP levels were statistically significant different between SCD and AD (*p*=0.003), and between MCI and AD (*p*=0.032). Plasma NfL was different in SCD vs MCI (*p*=0.026), SCD vs AD (*p*<0.001), SCD vs AD FU (*p*<0.001), SCD FU vs AD (*p=0.033*), SCD FU vs AD FU (*p=0.011*), MCI vs AD (*p*=0.002), MCI FU vs AD (*p*=0.003), MCI FU vs AD FU (*p*=0.003) and MCI vs AD FU (*p*=0.003). Plasma pTau 181 concentration was significantly different between SCD and AD (*p*=0.001), MCI and AD (*p*=0.026), MCI FU and AD (*p*=0.020). In APOE ϵ4 carriers, a statistically significant increase in plasma NfL (*p<0.001*) and pTau 181 levels was found (*p=0.014).* Moreover, an association emerged between age at disease onset and plasma GFAP (p = 0.021) and pTau181 (p < 0.001) levels.

**Discussion and conclusions:**

Plasma GFAP, NfL and pTau 181 are promising biomarkers in the diagnosis of the prodromic stages and prognosis of dementia.

## 1 Introduction

Alzheimer’s Disease (AD) is a neurodegenerative disorder and the most common form of dementia worldwide (1). The hallmark of AD is the deposition of Amyloid Beta (Aβ) plaques and neurofibrillary tangles (NFT) in neurons due to the hyperphosphorylation of tau protein, that leads to a growing neuronal death with a consequent gradual cognitive decline and impaired activities of daily life (2). A long prodromic phase, before first symptoms of dementia appear, was identified, characterized by two different and progressive stages: Subjective Cognitive Decline (SCD) and Mild Cognitive Impairment (MCI). According to the National Institute of Aging-Alzheimer’s Association (NIA-AA), SCD is the first manifestation of AD, defined as a self-perception of decline in memory and/or other cognitive abilities compared to the subject’s previously normal level of performance without any objective neuropsychological deficits (3, 4). MCI is a predementia stage with a presentation of cognitive impairment on standardized tests (4, 5). The currently recognized disease biomarkers of AD, such as PET neuroimaging or CSF biomarkers, are expensive and invasive techniques and are requested only when pathological changes are considerable. Biomarkers analyzed on blood sample are desirable as easily accessible and non-invasive tools. Plasma biomarkers are promising candidates for the early diagnosis of AD, tied to the biological β Amyloid (A) deposition, pathologic Tau (T), and Neurodegeneration (N) [ATN] framework (4, 6–8). Recently, plasma glial fibrillary acidic protein (GFAP), an intermediate filament protein of astrocytes, has been demonstrated to be associated with brain amyloid status and the development of clinical AD and cognitive decline (9, 10). Plasma neurofilament light chain (NfL), a cytoplasmic protein, is a marker of neurodegeneration associated with cognitive decline, brain atrophy, and hypometabolism (11–13). Plasma phosphorylated-tau (pTau) 181 was found to reflect tau pathological changes and was associated with tau positivity on positron emission tomography (PET) (14, 15). Moreover, clinical studies reported that plasmatic concentrations of GFAP, NfL and pTau 181 were correlated with the concentrations of the same biomarkers in the CSF, being increased in AD patients compared to Healthy Controls (HC) (16). Unfortunately, nowadays, plasma biomarkers, taken individually, are not sufficient to predict and/or make a defined diagnosis of AD. In addition, few studies have investigated the roles of plasma GFAP, NfL, and pTau 181 in the early stages of AD (17, 18).

Here, we evaluated the role of plasma GFAP, NfL, and pTau 181, combined together, as biomarkers for the early diagnosis of AD and for the assessment of progression to dementia.

## 2 Materials and methods

### 2.1 Patients

A total of 152 patients were consecutively recruited at the Neurology Unit of Careggi Hospital in Florence from September 2018 to May 2023. At first visit, all patients underwent a comprehensive clinical and neurological assessment. Forty-two patients received a clinical diagnosis of SCD (19), 74 of MCI (20) and 36 of AD (21). A blood sample for plasma and genetic analysis was collected at baseline for all patients, and a second plasma sample was isolated at follow-up visit, after two years, for 43 patients. During the first visit, a lumbar puncture was performed for CSF collection to analyze Aβ42, Aβ42/Aβ40, total‐tau (t‐tau) and p‐tau biomarkers. CSF samples were collected from 32 SCD patients, 66 MCI patients, and 34 AD patients. After collection, the CSF sample was immediately centrifuged and stored at −80 °C until testing. CSF biomarkers were measured using a chemiluminescent enzyme immunoassay (CLEIA) analyzer LUMIPULSE G600 (Fujirebio, Tokyo, Japan). Cut-off values for CSF were determined by following Fujirebio guidelines and normal values were: Aβ42> 670 pg/mL, Aβ42/Aβ40 ratio > 0.062, t‐tau < 400 pg/mL and p‐tau < 60 pg/mL (22).

According to the ATN system (4) and considering only the CSF analysis, we classified patients as: A+ = CSF amyloid biomarkers (Aβ42, Aβ42/Aβ40) lower than the cut-off values; A- = normal values of CSF amyloid biomarkers; T+ = CSF p-tau higher than the cut-off value; T- = CSF p-tau lower than the cut-off value; N+ = CSF t-tau higher than the cut-off value; N- = normal value of CSF t-tau. Therefore, we considered as “CSF +”, all the patients that had the following biomarker profiles: A+/T+/N+, A+/T-/N-, A+/T-/N+ and A-/T+/N+. In the other cases, patients were classified as “CSF –”.

Progression to MCI and AD was established according to the NIA‐AA criteria (20, 21). Age at baseline was the age at the time of plasma collection. A positive family history was defined as one or more first degree relatives with documented cognitive decline. The study protocol was approved by the local ethics committee and conducted in accordance with the provisions of the Helsinki Declaration.

### 2.2 Plasma analysis

Plasma was isolated from peripheral blood sample within 2 hours from collection. Blood sample was centrifuged at 4° at 1300 rcf for 10 minutes. The supernatant was immediately collected and stored at -80° until tested. Plasma analysis was performed on the automated Single molecule assay (Simoa) SR-X platform (Quanterix corp.). For GFAP and NfL measurement, the Simoa Human Neurology 2-Plex B assay (N2PB) (Item 103520) was used. The assay range of GFAP was 0 - ~40000 pg/mL, instead of NfL was 0 - ~2000 pg/mL. The N2PB kit analytical Lower Limit of Quantification (LLOQ) was for GFAP 4.15 pg/mL (pooled CV 16%, mean recovery 101%; Functional LLOQ = 16.6 pg/mL) and for NfL 0.400 pg/mL (pooled CV 18%, mean recovery 101%; Functional LLOQ = 1.60 pg/mL). The N2PB kit Limit of Detection (LOD) was 0.410 pg/mL (range 0.133-0.740 pg/mL) for GFAP and 0.071 pg/mL (range 0.012-0.149 pg/mL) for NfL measurements. Quality controls (1= low protein level, 2= high protein level) were included in the run (GFAP control 1= 94.8 pg/mL, control 2= 12701 pg/mL; NfL control 1= 4.21 pg/mL, control 2= 556 pg/mL). pTau 181 was detected using the Simoa® pTau-181 Advantage V2.1 Kit (Item 104111). The Analytical LLOQ of the pTau 181 kit was 2.23 pg/mL (pooled CV 19.0%, mean recovery 97.0%; Functional LLOQ =8.92 pg/mL), instead the LOD was 1.04 pg/mL (range 0.146-1.750 pg/mL). pTau 181 quality controls were: control 1= 50.5 pg/mL, control 2= 530 pg/mL. The N2PB and pTau 181 kit analysis in all samples were performed in a single run basis, respectively. All plasma samples were diluted to 1:4. A reference calibration curve was established using serially diluted calibrators, provided by Quanterix. Plasma samples, calibrators, and controls were run in duplicate.

### 2.3 Apolipoprotein E genotyping

Genomic DNA was isolated from peripheral blood samples with the QIAamp DNA blood mini QIAcube Kit (Qiagen, Germany), using the automatized extractor Qiacube, provided by Qiagen Corp, following the manufacturer protocol. DNA amount and quality was checked with the QIAxpert spectrophotometer (Qiagen, Germany). APOE genotypes were investigated using two sets of PCR primers designed to amplify the regions encompassing rs7412 [NC_000019.9:g.45412079C > T] and rs429358 (NC_000019.9:g.45411941T > C) and PCR products were analyzed by the high-resolution melting analysis (HRMA) (23). Control samples with known APOE genotypes, validated by DNA sequencing, were used as standard references.

### 2.4 Statistical analysis

Statistical analysis was performed using SPSS software version 28 (IBM SPSS Statistics). Continuous variables were correlated using Pearson’s correlation analysis. Shapiro–Wilk test was used to test the normal distribution of data. To analyze differences between groups, we used independent-samples t-test, Mann-Whitney U test, and Kruskal Wallis test and ANOVA test for differences between more than two groups. Welch t-test was performed when the assumption of homogeneity of variances was violated. To test whether the difference between two proportions is statistically significant we used Fisher's exact test. p < .05 was set as significant. Continuous variables were reported as mean ± standard deviation. Allele frequency of APOE ϵ4 was determined by counting and calculating sample proportions.

## 3 Results

Clinical and demographic data of 152 patients (42 SCD, 74 MCI and 36 AD) are shown in **Table 1**. A difference was present between groups in mean age at disease onset (SCD vs MCI, β=-3.396, *p<0.001*; SCD vs AD, β=-3.486, *p<0.001*; not between MCI vs AD; *p=0.344*), with a later onset in AD patients (67.22 ± 6.536; mean ± SD) compared to MCI (65.47 ± 8.124) and SCD (59.38 ± 8.403). A statistically significant difference in APOE ϵ4 allele distribution was found between groups (MCI vs AD β=-2.442 *p=0.015*; SCD vs AD β=-3.163 *p=0.002*, not between SCD vs MCI, *p=0.214*), as AD patients showed a higher ϵ4 allele percentage (33.3%) than MCI (23.6%) and SCD (15.4%).

**Table 1:** Clinical and demographic data of 152 studied patients.

	SCD	MCI	AD
***n***	42	74	36
**Sex f/m**	28/14	48/26	19/17
**Age at onset *mean (SD)***	59.38 (8.403)	65.47 (8.124)	67.22 (6.536)
**APOE ϵ4 allele frequency *(%)***	13/84 (15.4)	35/148 (23.6)	24/72 (33.3)
**CSF + biomarker frequency *(%)***	7/32 (21.8)	35/66 (53)	30/34 (88.2)

CSF+ positive biomarker, when A+/T+/N+, A+/T-/N-, A+/T-/N+ and A-/T+/N+ (A+=CSF amyloid biomarkers (Aβ42, Aβ42/Aβ40) lower than the normal values; A- = normal values of CSF amyloid biomarkers; T+ = CSF pospho-tau higher than the normal value; T- = normal value of CSF pospho-tau; N+ = CSF t-tau higher than the normal value; N- = normal value of CSF t-tau).

AD, Alzheimer’s Disease; APOE, Apolipoprotein E; CSF, Cerebrospinal fluid; f, females; m, males; n, number; MCI, Mild Cognitive Impairment; SCD, Subjective Cognitive Decline; SD, Standard Deviation.

**Figure 1:**
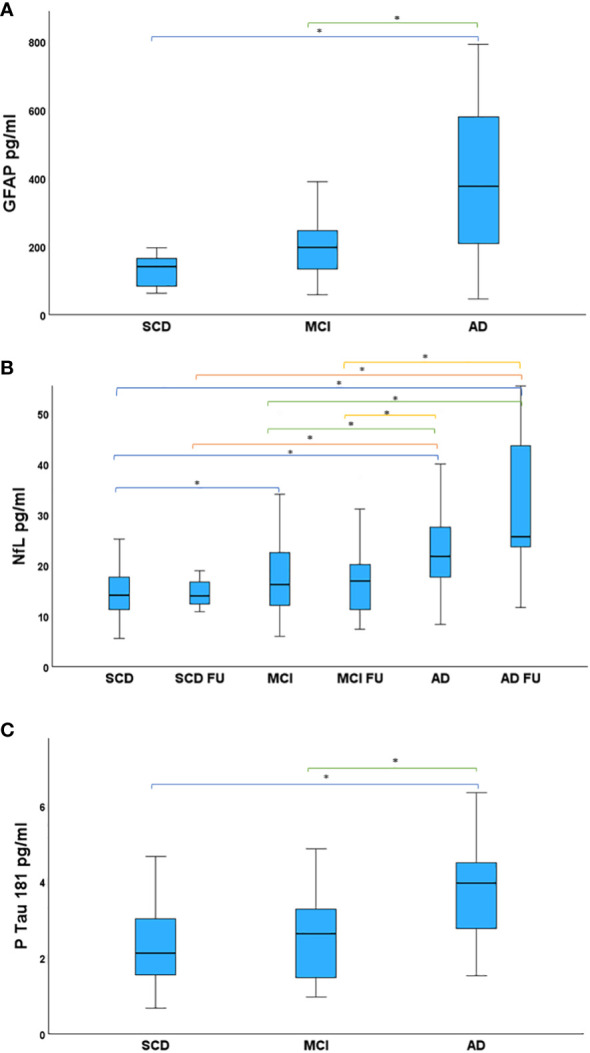
Study patients, divided in groups, compared for mean plasma biomarker levels. **(A)** Mean GFAP levels in the studied groups. AD showed higher GFAP levels than MCI and SCD. **(B)** Mean NfL levels in the studied groups. NfL levels were highest in AD FU. AD had higher NfL concentration in plasma than SCD, SCD FU, MCI, MCI FU. NfL in MCI were increased with respect to SCD. **(C)** Mean pTau 181 levels in the studied groups. Statistical significance accepted at the p < .05, highlighted with asterisk. AD had increased pTau 181 concentration compared to MCI and SCD. AD, Alzheimer’s Disease; FU, Follow-Up; GFAP, Glial Fibrillary Acidic Protein; MCI, Mild Cognitive Impairment; NfL, Neurofilament Light Chain; pTau 181, phosphorylated Tau 181; SCD, Subjective Cognitive Decline.

Follow-up (FU) plasma samples were available for 43 patients: 7 SCD, 27 MCI and 9 AD. Plasma NfL measurement was executed both on 152 plasma samples collected during the first visit and on the 43 FU samples. Instead, GFAP and pTau 181 were analyzed, at a later time, on aliquots of plasma samples of the first visit still available: GFAP was measured in 42 plasma samples (5 SCD, 25 MCI, 12 AD); pTau 181 was measured in 81 plasma samples (25 SCD, 39 MCI, 17 AD). Data analysis of plasma measurements of GFAP, NfL and pTau 181 are shown in **Figure 1**. GFAP levels in plasma samples were significantly different between SCD and AD (β=-3.379, *p<0.001*) and between MCI and AD (β= -2.314, *p=0.021*), but not between SCD and MCI (*p=0.054*). AD patients showed an increased GFAP concentration in plasma (391.10 ± 245.90) with respect to MCI (219.21 ± 103.10) and SCD (128.31 ± 63.67) (**Figure 1A**). Statistical differences in plasma NfL levels between each group are shown in **Table 2**. In particular, SCD had lower NfL levels (14.69 ± 6.07) compared to other groups: SCD FU (17.05 ± 9.02), MCI (18.51 ± 9.80), MCI FU (17.06 ± 7.02), AD (27.61 ± 25.09) and AD FU (33.41 ± 16.88) (**Figure 1B**). A statistically significant linear relationship emerged between diagnosis and mean of NfL concentration (F(1,193)= 21.635, *p<0.001*, R^2^ = 0.102). Plasma pTau 181 concentrations were significantly different between SCD and AD (β*= -*3.320*, p<0.001*) and between MCI and AD (β*=-*2.914*, p=0.004*). In fact, AD had higher pTau 181 levels (3.81 ± 1.44) than MCI (2.69 ± 1.27) and SCD patients (2.17 ± 1.02) (**Figure 1C**).

**Table 2:** Pairwise comparisons of plasma NfL levels between groups.

	B	*SE*	β	p-value
**SCD-SCD FU**	-11,369	22,804	-,499	,618
**SCD-MCI FU**	-18,163	13,779	-1,318	,187
**SCD-MCI**	-24,125	10,818	-2,230	,026*
**SCD-AD**	-60,569	12,784	-4,738	,000*
**SCD-AD FU**	-83,163	20,518	-4,053	,000*
**SCD FU-MCI FU**	-6,794	23,692	-,287	,774
**SCD FU-MCI**	-12,756	22,102	-,577	,564
**SCD FU-AD**	-49,200	23,128	-2,127	,033*
**SCD FU-AD FU**	-71,794	28,150	-2,550	,011*
**MCI FU-MCI**	5,963	12,582	,474	,636
**MCI FU-AD**	-42,406	14,308	-2,964	,003*
**MCI FU-AD FU**	-65,000	21,500	-3,023	,003*
**MCI-AD**	-36,444	11,484	-3,173	,002*
**MCI-AD FU**	-59,037	19,734	-2,992	,003*
**AD-AD FU**	-22,594	20,877	-1,082	,279

Between-group comparisons: ANOVA with Bonferroni post-hoc. Statistical significance accepted at the p < .05, highlighted with asterisk. AD, Alzheimer’s Disease; FU, Follow Up; MCI, Mild Cognitive Impairment; NfL, Neurofilament Light Chain; SCD, Subjective Cognitive Decline; SE, Standard Error.

A statistically significant positive association was found between APOE ϵ4 carriers and NfL levels (β=11.132, *p<0.001*), and between APOE ϵ4 carriers and pTau 181 levels (β*=*6.040, *p=0.014*). APOE ϵ4 carriers had higher NfL (23.11±19.16) and pTau181 levels (3.10 ±1.20) than non-carriers (NfL=16.89±7.62; pTau 181=2.51±1.32). No differences emerged in biomarker levels between APOE ϵ4 homozygous and heterozygous carriers. Moreover, a positive correlation emerged between age at disease onset and plasma GFAP (β = 0.372, p = 0.021) and pTau 181 (β = 0.439, p < 0.001) levels.

Patients with a higher age at disease onset had increased plasma levels of GFAP and pTau 181.

CSF analysis was performed in 132 patients (32 SCD, 66 MCI, and 34 AD), and a statistically significant association between CSF+ biomarkers and diagnosis was found (β=27.762, *p<0.001*). AD patients had a higher frequency (88.2%) of CSF+ biomarkers than MCI (53%) and SCD (21.8%) patients. A comparison between patients with CSF+ biomarkers and patients with negative CSF biomarkers highlighted a significant difference in mean age at disease onset (67 ± 5.93 vs 60.98 ± 9.33 years, 95%CI 2.977-9.061, *p<0.001*), in mean GFAP levels (350.08 ± 194.19 vs 158.75±88.43 pg/ml, 95%CI 89.63-293.01, *p<0.001*), in mean NfL levels (24.86 ± 19.91 vs 14.34 ± 5.26, 95%CI 5.27-15.75, *p<0.001*), in mean pTau 181 levels (3.66 ±1.25 vs 2.02 ± 0.87, 95%CI 1.11-2.15, *p<0.001*). No difference in APOE ϵ4 frequency between the two groups was found.

## 4 Discussion

The aim of our study was to investigate plasma biomarkers in a cohort of patients with different stage of cognitive decline and to evaluate if they could represent a diagnostic tool. In particular, we evaluated GFAP, NfL and pTau 181 in AD and in its preclinical stages (SCD and MCI). Our results showed that these plasma biomarkers were able to distinguish the different phases of the disease. Increasing plasma biomarker levels correlated to the progress and stage of the disease. In fact, AD patients had higher plasma levels of GFAP, NfL and pTau 181 with respect to MCI, and with respect to SCD that had lowest concentrations. Patients with CSF + biomarkers for AD pathology exhibited increased levels of plasma biomarkers. A greater age at disease onset correlated with a higher plasma concentration of GFAP and pTau 181. These results provide evidence that plasma biomarkers can detect advancing AD-associated biologic changes, discriminating all the stages of AD continuum and predicting cognitive decline. For these reasons, GFAP, NfL and pTau 181 can be useful tools to support clinical diagnosis.

Moreover, we tested thepower of plasma NfL in relation to the progression of the disease. A FU plasma sample was available for a subgroup of patients and data highlighted the capability of NfL to assess the longitudinal changes along the AD continuum. There was a linear association between the dementia stage and mean NfL levels. In fact, SCD showed the lowest NfL levels while AD FU showed the highest value of this biomarker. This study extended and confirmed our previous results, where we speculated that plasma NfL can predict the underlying AD pathology (24–26). Our previous data showed that patients who progressed through the dementia stages had higher plasma NfL levels than non-progressive patients. Moreover, we identified plasma NfL cut-off values of 19.45 pg/mL for SCD and 20.45 pg/mL for MCI (25). Our results were in line with literature data (27–29). Simrén and colleagues established the plasma NfL reference limit value at 20 pg/mL for the neurologically healthy individuals (29). Furthermore, studies conducted on AD mutation carriers reported that plasma NfL can predict AD 16 years before symptom manifestation, increasing in the transition from the preclinical to clinical phase (30, 31).

Our findings corroborate data from previous studies that reported elevated plasma GFAP, NfL and pTau181 in preclinical AD, prodromal AD, and AD (7–9, 15, 32–39). Taking into consideration all the plasma biomarkers together, they can provide a plasma profile of dementia to aid clinical assessment and identify different clinical phenotypes. Plasma-based biomarkers are a better choice because of their simple, easily accessible, repeatable, and inexpensive characteristics. More importantly, the possibility of recognizing the preclinical phase is fundamental for clinical decision-making and more efficient management of the patient (40, 41). The goal of early diagnosis is linked to the early therapeutic strategy (42, 43).

In addition, our studied patients carrying the genetic AD risk factor, ϵ4 allele of APOE, had higher NfL and pTau 181 levels than non-carriers. Since 2004 studies have suggested that APOE genotype modulates NFT development and Aβ metabolism (44, 45). APOE ϵ4 allele is thought to be involved in Aβ and neuritic plaque accumulation, affecting Aβ clearance (46–48). *In vivo* experiments on AD transgenic mouse models showed differences in Aβ deposition in an ApoE isoform-dependent manner: mice ApoE ϵ4-expressing had more than 10-fold fibrillar deposits compared to ApoE ϵ3 and ApoE ϵ2 mice (46, 49). Both *in vitro* and animal experiments suggest that ApoE ϵ4 promote NFT inclusions, inducing the activation of glycogen synthase kinase (GSK) 3β, an enzyme responsible for the tau hyperphosphorylation (50–52). Thus, plasma NfL and pTau 181, combined with APOE genotype, may help to identify individuals at increased risk of dementia.

This study has several limitations: i) The number of patients was relatively small. ii) A control group was not present to verify that levels of plasma biomarkers in healthy individuals are lower than in preclinical stages, thus all plasma biomarker levels were compared to literature data. iii) The plasma GFAP and pTau 181 data were limited with respect to the total sample size as plasma aliquots were not available for all. iv) A FU plasma sample was available only for a subgroup of patients (43) and a longitudinal study was possible only for NfL, the first peripheral protein evaluated by our team.

On the other hand, all samples were collected prospectively, processed and stored using the same standardized method and measurements of plasma biomarkers were done in a single batch, ensuring good reproducibility. Moreover, few studies have investigated the combination of GFAP, NfL, and pTau 181 in the preclinical stage of dementia (17, 18).

In conclusion, our study provides evidence for the potential use of plasma biomarkers in the diagnosis and prognosis of preclinical stages of dementia. Plasma GFAP, NfL and pTau 181 can distinguish all phases along the AD continuum. Their levels increase mirroring the progression of the neurodegeneration. The measurement of peripheral proteins through a simple and non-invasive blood sample is desirable for clinical routine and for patient management. Moreover, in future we will extend data analysis on GFAP and pTau 181 in all the samples in order to confirm our findings.

## Data availability statement

The original contributions presented in the study are included in the article/supplementary materials, further inquiries can be directed to the corresponding author/s.

## Ethics statement

The studies involving humans were approved by the local ethics committee and conducted in accordance with the provisions of the Helsinki Declaration. The studies were conducted in accordance with the local legislation and institutional requirements. The participants provided their written informed consent to participate in this study.

## Author contributions

AI: Writing – review & editing, Writing – original draft. SB: Writing – review & editing. SM: Writing – review & editing. GG: Writing – review & editing. VB: Writing – review & editing. CF: Writing – review & editing. SS: Writing – review & editing. BN: Writing – review & editing.
